# Prediction of gene–phenotype associations in humans, mice, and plants using phenologs

**DOI:** 10.1186/1471-2105-14-203

**Published:** 2013-06-21

**Authors:** John O Woods, Ulf Martin Singh-Blom, Jon M Laurent, Kriston L McGary, Edward M Marcotte

**Affiliations:** 1 Center for Systems & Synthetic Biology, Institute for Cellular & Molecular Biology, The University of Texas at Austin, Austin, TX 78712, USA; 2 Program in Computational and Applied Mathematics, The University of Texas at Austin, Austin, TX 78712, USA; 3 Unit of Computational Medicine, Department of Medicine, Karolinska Institutet, Stockholm 171 76, Sweden; 4 Department of Biological Sciences, Vanderbilt University, Nashville, TN 37235, USA

## Abstract

**Background:**

Phenotypes and diseases may be related to seemingly dissimilar phenotypes in other species by means of the orthology of underlying genes. Such “orthologous phenotypes,” or “phenologs,” are examples of deep homology, and may be used to predict additional candidate disease genes.

**Results:**

In this work, we develop an unsupervised algorithm for ranking phenolog-based candidate disease genes through the integration of predictions from the *k* nearest neighbor phenologs, comparing classifiers and weighting functions by cross-validation. We also improve upon the original method by extending the theory to paralogous phenotypes. Our algorithm makes use of additional phenotype data — from chicken, zebrafish, and *E. coli*, as well as new datasets for *C. elegans* — establishing that several types of annotations may be treated as phenotypes. We demonstrate the use of our algorithm to predict novel candidate genes for human atrial fibrillation (such as *HRH2*, *ATP4A*, *ATP4B*, and *HOPX*) and epilepsy (e.g., *PAX6* and *NKX2-1*). We suggest gene candidates for pharmacologically-induced seizures in mouse, solely based on orthologous phenotypes from *E. coli*. We also explore the prediction of plant gene–phenotype associations, as for the *Arabidopsis* response to vernalization phenotype.

**Conclusions:**

We are able to rank gene predictions for a significant portion of the diseases in the *Online Mendelian Inheritance in Man* database. Additionally, our method suggests candidate genes for mammalian seizures based only on bacterial phenotypes and gene orthology. We demonstrate that phenotype information may come from diverse sources, including drug sensitivities, gene ontology biological processes, and *in situ* hybridization annotations. Finally, we offer testable candidates for a variety of human diseases, plant traits, and other classes of phenotypes across a wide array of species.

## Background

Computational prediction of complex phenotypes from underlying genes has largely involved increasingly complex *in silico* simulations of cells and cellular processes. Last year, for example, Karr et al. published a whole-cell computational model made up of twenty-eight submodels, each a simulation of a specific cellular process [1]. Most methods are variations on flux–balance analysis for predicting metabolic phenotypes [2], in most cases including transcriptional regulatory information [3-6], and yield primarily quantitative data.

In contrast, a number of qualitative methods make use of guilt-by-association in functional networks to predict gene–phenotype associations (reviewed in [7,8]). Like the quantitative methods, these network-based techniques are species-specific, though they may incorporate data from additional species. While quantitative methods are limited to unicellular organisms, or at least to unicellular phenotypes of multi-cellular organisms, the qualitative methods can provide insight into whole-organism traits.

**Figure 1:**
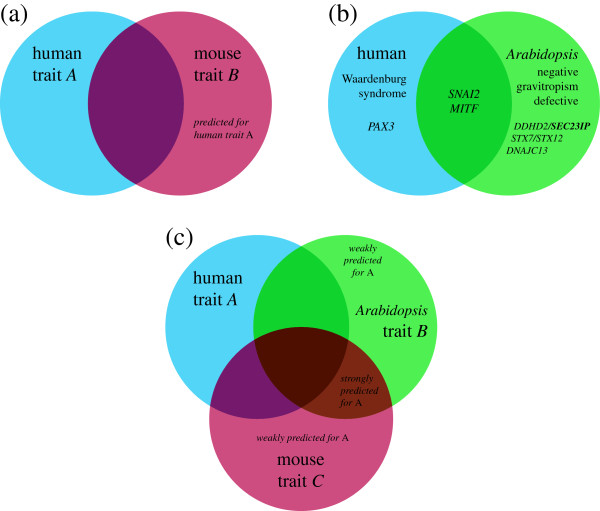
**Prediction of disease–genes from orthologous phenotypes. (a)** Two phenotypes are said to be orthologous (“phenologs”) if the sets of underlying genes for those phenotypes have a statistically significant intersection, as determined using gene orthology. Statistical significance is calculated as the probability of seeing an intersection of *v* or greater given *m* genes with phenotype *A* and *n* with phenotype *B*, out of *N* total genes with orthologs in both species. Genes associated with *A* but not *B* are said to be predicted to be involved with *B*, and vice-versa. McGary et al. observed that approximately *v*/*m* of the predictions tended to be true positives for *B*, and *v*/*n* to be true positives for *A*. **(b)** illustrates a validated example from McGary et al. predicting genes involved in a human neural crest defect, Waardenburg syndrome, using the *Arabidopsis* negative gravitropism defect phenotype. In this example, the overlap between gene sets affiliated with Waardenburg and gravitropism is highly statistically significant (*p*≤10^−6^). In the right-hand circle and intersection, the human orthologs of the gravitropism genes are shown, for simplicity (*VAM3* corresponding to *STX7*, *STX12*; *SGR2* to *DDHD2*, *SEC23IP*; and *GRV2* to *DNAJC13*). **(c)** In this paper, we extend the phenolog formalism to consider additional gene–phenotype associations from multiple model organisms to develop a quantitative ranking scheme for phenolog-based predictions. Those genes predicted by a single phenolog, as in **(a)**, are *weakly predicted for A*; whereas those predicted by two phenologs are *strongly predicted for A*. In general, the addition of a third phenolog contributing to a predicted association will cause that gene to be ranked higher than if only two phenologs predict it. However, not all phenologs are equal; phenologs derived from less similar gene sets exert less influence over predictions than phenotypes with highly overlapping sets of affiliated genes.

In 2010, McGary et al. described a separate qualitative method which relies on orthology rather than gene networks. Specifically, human traits, diseases, and phenotypes may have orthologous properties in other organisms, and such properties — typically phenotypes — are identifiable based on orthology of the underlying genes. Such orthologous phenotypes, or phenologs, can be used to predict novel disease-causing genes as in the manner summarized in Figure 1. For example, McGary et al. identified *SEC23IP* as a neural crest effector, potentially involved in Waardenburg syndrome, based on its association with negative gravitropism defects in *Arabidopsis*[9].

Phenologs are a natural extension of the concept of deep homology: as a bird’s wing and a human hand arose from a common ancestor structure with a common complement of genes and a similar developmental program [10], so also might less obviously related phenotypes derive from a common ancestor phenotype affiliated with an underlying conserved gene module. To take the above example of Waardenburg syndrome, certain mammalian neural crest defects and plant gravitropism defects share and partly arise from an ancient, highly conserved vesicle trafficking system.

We set out to improve upon the original phenolog algorithm, which relies on identifying pairs of matching phenotypes across species, with a goal of ranking candidate genes relevant to specific traits and diseases by way of an unsupervised search for similar phenotypes (Figure 1). We reasoned that gene–phenotype association predictions coming from multiple “nearby” (or high similarity) phenologs, preferably across multiple species, should provide more predictive power than those from single phenologs. Our method ranks candidate genes based on both the number and similarity of cognate phenotypes which involve those genes, which might be used as a prioritization for wet lab experiments (Figure 1C).

Additionally, we expanded upon the original phenolog study — which included gene–phenotype data from human, mouse, worm (*C. elegans*), baker’s yeast, and *Arabidopsis thaliana* — by adding data from chicken, zebrafish, and even *E. coli*, as well as additional human and worm datasets. We show that phenotype data may come from a variety of sources, including GO biological processes and gene tissue expression annotations, and that the integration of signal from multiple phenologs markedly improves the predictive power of the method.

A key advantage to a neighborhood-based approach for predicting gene–phenotype associations is the ease with which non-obvious — and thus interesting — biological stories may be teased out. We demonstrate the process with epilepsy, a human syndrome; mouse susceptibility to pharmacologically-induced seizures, a related phenotype, using only *E. coli* data; and atrial fibrillation, the leading cause of arrhythmia in humans.

In addition to offering concrete predictions, we compared two classifiers for integrating phenologs (additive and naïve Bayes), across a variety of similarity or distance functions, and with different numbers of neighboring phenotypes (*k*). We also experimented with changing the weighting function used to assign prediction scores, and we tested two frameworks for translating gene–phenotype associations between species, evaluating all of these methods within a consistent cross-validation scheme.

## Results and discussion

### A matrix-based formalism for comparing gene–phenotype associations between species

The phenolog approach, developed by McGary et al. in [9], identifies *pairs* of homologous phenotypes in different organisms by counting the overlap between the sets of genes associated with them. McGary et al. hypothesize that pairs of phenotypes with a greater than expected number of shared genes derive from a shared evolutionary past, and further hypothesize that genes associated with one might therefore be good candidates for the other. To extend this conceptual framework to make predictions based on multiple phenotypes from multiple species, we developed a matrix-based formalism for integrating phenotypic information.

For a given species, the set of all gene–phenotype associations can be thought of as a matrix where rows correspond to genes and columns to phenotypes, and the matrix has a 1 in position *i*,*j* if the *i*-th gene has been observed to be associated with the *j*-th phenotype. This formalism works well when all the genes and phenotypes studied are from the same organism, but leads to some complications when extended to pairs or groups of species.

In particular, since generating these gene–phenotype matrices involves translation via gene orthology, we investigated whether expansions and contractions of gene families (e.g., in *Arabidopsis*, which frequently has large paralogous gene expansions relative to other eukaryotes) might produce enough noise to obscure signal from other gene–phenotype associations of interest.

**Figure 2:**
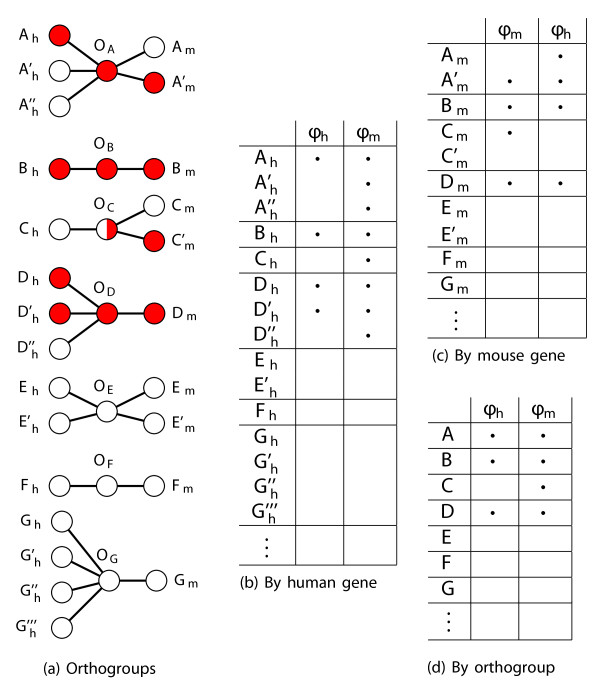
**The matrix formalism for calculating phenolog overlaps is especially important when predicting between species where large gene family expansions have occurred since species divergence, such as between *Arabidopsis *and humans.** The example uses human and mouse to illustrate the orthogroup-based matrix formalism. **(a)** Phenotype associations (colors) are plotted as graphs for genes from human (left nodes, subscripted *h*) and mouse (right nodes, subscripted *m*), showing genes’ orthology relationships (edges radiating from orthogroups — middle nodes, labeled *O*). The orthologies (from INPARANOID), are used to “translate” phenotype associations between species (in the case of the gene-based matrix framework in panels **(b, c)**) or into an intermediate collection of orthogroup–phenotype associations (for the orthogroup-based matrix framework in **(d)**). Orthogroup vertices (e.g., *O*_*A*_) connect human and mouse orthologs (such as *A*_*h*_, *Ah*′, and *Ah*″, which are paralogs of one another relative to the human–mouse divergence, with *A*_*m*_ and *Am*′. Red vertices within a species are genes associated with the phenotype of interest (*ϕ*_*h*_ for human and *ϕ*_*m*_ for the mouse phenotype); orthogroup colors reflect the species data. These associations can alternately be captured by representing the graphs as matrices **(b–d)**, with bullets indicating an assocation between a given genetic element and a phenotype. Specifically, **(b)** and **(c)** represent the gene-based formalism, and **(d)** illustrates the orthogroup-based formalism. Human and mouse phenotype columns are indicated by *ϕ*_*h*_ and *ϕ*_*m*_, respectively.

In order to address this question, we developed two different frameworks within our matrix formalism for translating gene–phenotype associations between species (Figure 2). In the first, the “gene-based” approach, we let the rows correspond to genes in the species that we wish to make predictions for, and translated the gene–phenotype interactions from a number of species by orthology. This gave us a number of species-specific gene–phenotype association matrices *Φ*_S_, for S∈{human, mouse, yeast, nematode, plant, zebrafish, fly, chicken }, where each *Φ*_S_ is defined by

$${\left(\Phi_{S}\right)}_{\text{ij}}=\left\{\begin{array}{ll} 1 & \text{if any ortholog in S of gene}i\\ & \text{is associated with phenotype}j,\\ 0 & \text{otherwise.} \end{array}\right)$$

We used the INPARANOID algorithm [11] to determine which genes in different organisms are orthologs of each other. The INPARANOID algorithm discovers orthology relationships in the form of orthogroups (Figure 2A).

For the method described above, we simply translated other species’ gene–phenotype associations into the target (e.g., into human genes when predicting human gene–disease associations) gene–phenotype matrix by orthogroup, and compared the phenotype columns in terms of human genes (as in Figure 2B–C).

This gene-based approach works very well for closely-related species, where genes often have one-to-one equivalents between species. However, when large orthogroups are involved, the predictive performance of this approach deteriorates.

To mitigate the decrease in performance caused by paralogous gene expansions we devised an “orthogroup-based” matrix framework, in which rows corresponded to INPARANOID orthogroups (Figure 2A and D) rather than actual genes (Figure 2B–C),

$${\left(\Phi_{S}\right)}_{\text{ij}}=\left\{\begin{array}{ll} 1 & \text{if any gene in the orthogroup}i\\ & \text{is associated with phenotype}j,\\ 0 & \text{otherwise.} \end{array}\right)$$

Notably, the use of orthogroups can dramatically simplify the relationships. Consider orthogroup *O*_*A*_ (Figure 2A): one gene from each species is involved in the phenolog; whereas in Figure 2B, a matrix with each row representing a human gene, a single mouse gene–phenotype association translates into three human gene associations because of the paralogous expansion of this gene family in humans. Similarly, in Figure 2C, in which each row is a mouse gene, a single human gene–phenotype association translates into two mouse gene associations, due to a mouse-specific paralogous expansion. In contrast, the orthogroup-based matrix in Figure 2D permits a symmetric comparison of the phenotypes, reducing paralogs in each species to a single orthogroup. Furthermore, a hypergeometric CDF test of the intersection between phenotypes *ϕ*_*h*_ and *ϕ*_*m*_ will produce different values for the matrices described in Figures 2B–C. The consequence of asymmetric distances is that *ϕ*_*h*_ may have *ϕ*_*m*_ as its closest neighbor when the search is performed in one direction, but *ϕ*_*m*_ may not have *ϕ*_*h*_ as its closest neighbor in the reverse search.

The orthogroup-based matrix (Figure 2D) has the advantage of producing consistent, symmetric similarity scores regardless of the direction of prediction; furthermore, these scores are not inflated by the co-occurrence of multiple phenotype observations in a single orthogroup. Unless otherwise noted, we use this framework for the analyses that follow.

### Integrating information from multiple phenologs

Given this basic formalism — a matrix of gene–disease associations incorporating phenotypic data from multiple species — we next evaluated methods for ranking genes on the basis of their tendency to be involved in a phenotype of interest. In other words, we wanted to construct a set of predictions *X* for gene–phenotype associations such that *X*_*ij*_ is higher for pairs where the gene *i* is actually associated with the phenotype *j*.

One way to incorporate information from multiple phenotypes is by measuring the similarity — in terms of associated genes — between pairs of phenotypes, and integrating the information from different phenotypes in such a way such that more similar phenotypes get more weight than less similar phenotypes. We tested two different ways of integrating this information — one multiplicative naïve Bayes scheme, and one additive method.

The naïve Bayes scheme we used was first described in the original phenolog paper [9], and can be written as follows:

(1) $$\begin{array}{c} \;X_{\text{ij}}=P(\text{gene}i\in \text{disease}j|k\;\text{phenologs})=1-\overset{k}{\underset{l=1}{\prod }}\left(1-f_{\text{ijl}}w_{\text{jl}}\right) \end{array}$$

where

(2) $$\;\begin{array}{c} f_{\text{ijl}}=P(\text{gene}\;i\in \text{disease}j|\text{phenotypes}j\text{and}l\text{are phenologs}) \end{array}$$

(3) $$\;\begin{array}{c} w_{\text{jl}}=P(\text{phenotypes}j\text{and}l\text{are phenologs}) \end{array}$$

We tested a wide variety of measures for the weighting function *w*_*jl*_ that calculates a similarity or distance between two sets. Pearson sample correlation is a particularly popular option for expert recommendation systems, such as those used in online retail for generating recommendations from past purchase history. McGary et al. used the hypergeometric CDF, which gives the probability of seeing an intersection of size *v* or greater between phenotypes containing *m* and *n* genetic elements, with *N* total elements in the species pair (Figure 1A–B).

For *f*_*ijl*_ we used *v*/*n*, the fraction of the number of genes common to both phenotypes *j* and *l* over the number of genes known to be involved in phenotype *j*, which empirically appears to be a good approximation of the probability that a candidate gene from a single phenolog will turn out to be a true positive [9].

While the naïve Bayes method multiplies distances or similarities as if they were probabilities, for the additive method, *X*_*ij*_ is calculated for each gene–phenotype pair (*i*,*j*) by taking the sum over all nearest neighbor phenotypes *k*, weighted by the similarity between phenotypes *j* and *k*, so

(4) $$X_{\text{ij}}=\underset{k}{\sum }w_{\text{jk}}\Phi_{\text{ik}}={\left(\Phi w^{T}\right)}_{\text{ij}},$$

where *Φ* is a phenotype matrix and *w* is a weight matrix of phenotype–phenotype similarity scores.

**Figure 3:**
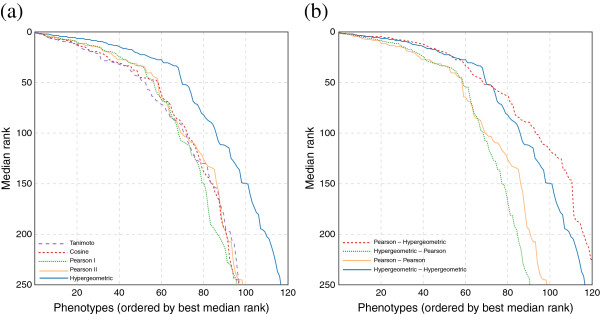
**Effect of distance measure choice for ordering and weighting phenotypes.** Here we plot for how many diseases the median rank of the gene withheld during leave-one-out cross-validation stays at a certain level, using all available species, and integrating the results using the naïve Bayes scheme. In **(a)**, we vary the distance and weighting function (using the same measure for both). In **(b)**, we show the effect of varying the distance function independently from the weighting function. Here the first function in the legend is the distance function used for computing the *k* nearest neighbors, and the second is the weighting function *w*_*ij*_ from Equations 1 and 4. As can be seen from the figure, a good distance function has more effect on performance than a good weighting function, but that the results can be improved slightly by using a combination: hypergeometric for distance, and Pearson for integration.

In addition to the hypergeometric CDF and Pearson sample correlation, we tested Euclidean distance, taxicab (Manhattan) distance, cosine distance and Tanimoto coefficient as measures of phenotypic similarity, both for finding the *k* nearest neighbors and as weighting functions. We expected that orthologous phenotypes from closely related species might show more similar gene sets than those from more distantly related species. In turn, various distance functions might account for this bias to a greater or lesser extent; we thus compared different distance functions using cross-validation, as noted later. Euclidean and Manhattan distance performed extremely poorly in the gene framework, using five-fold cross-validation, so we excluded them from analyses in the orthogroup framework. Overall, the Pearson coefficient and hypergeometric test appear to have the most power for identifying nearby predictive phenologs (Figure 3A).

We also repeated the analysis while varying the distance function (used for searching) and holding the recommendation function (*w*) the same, and vice-versa (Figure 3B). Pearson sample correlation showed the best performance among the distance functions; however, we found that the hypergeometric CDF was the best weighting function for assigning prediction scores to genes.

**Figure 4:**
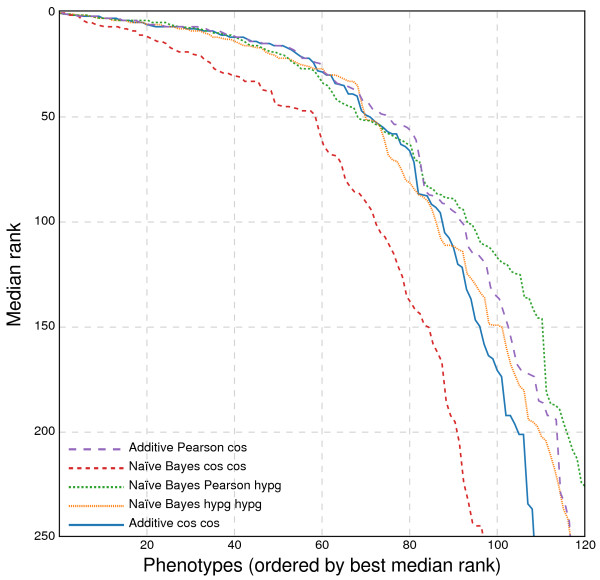
**Predictive performance of the orthogroup-based matrix approach.** Here we show a comparison of naïve Bayes and additive classifier predictions, which seem to have similar performance, using leave-one-out cross-validation. As in Figure 3B, the first function in the legend is the distance function used for computing the *k* nearest neighbors, and the second is the weighting function *w*_*ij*_ from Equations 1 and 4.

We compared the naïve Bayes and additive classifiers, with the results shown in Figure 4. The performance in cross-validation is quite similar between the two classifiers, with the best version of the naïve Bayes classifier (using Pearson sample correlation for distance and hypergeometric CDF for weighting) performing slightly better than the best additive one (using Pearson sample correlation and hypergeometric CDF). However, the additive classifier allows us to visualize and deconstruct the predictions into component phenotypes. We therefore chose to use the additive classifier for most predictions.

**Figure 5:**
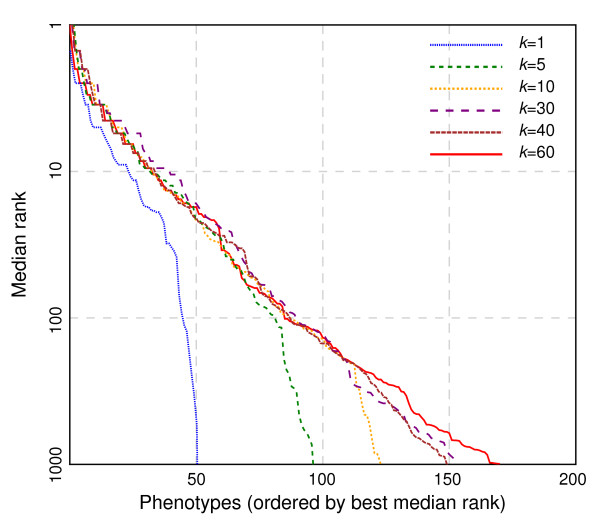
**Effect of *k *on predictiveness.** Using the same cross-validation setup as in Figure 4, we compare different *k*-values in the neighborhood search for phenologs. Any *k* greater than 1 gives a great improvement in the high-precision regime. However, as *k* increases further, the improvements in the recovery affect successively less important ranks, with diminishing returns as *k* approaches 30.

Varying the maximum number of neighbors (*k*) tends to affect lower-ordered predictions (e.g., the thousandth gene predicted for a disease) to a larger extent than top predictions. Figure 5 shows that even including the *k*=5 nearest neighbors improves the results modestly — raising the number of diseases for which the withheld genes can be predicted at a top-100 median rank from around 50 to 80. Searching for the *k*=40 nearest neighbors seems to offer no meaningful improvement over *k*=10 at relevant ranks. Thus, while a higher value of *k* may not always provide the best predictor, it is more likely, on average, to be useful than only considering the single closest phenotype.

Some phenotypes were intrinsically unpredictable; notably, several of these were revealed to be combinations of unrelated diseases that were overcollapsed into the same entity in the initial version of our OMIM database (two such examples were achromatopsia with achondroplasia, and the combination of all blood type genes), thus serving inadvertantly as negative controls.

The best similarity functions produced highly correlated predictions. Further, the best predictions of the worst classifiers were highly correlated with the best predictions of the top-performing classifiers. We thus concluded that the potential benefits of a fusion or blending classifier, a model that draws the best characteristics from simple classifiers via optimization, would be modest at best. At worst, any improvement would be difficult to measure; optimization of such a blending model would require an additional layer of cross-validation, and many phenotypes would need to be dropped due to relatively small associated gene sets.

**Figure 6:**
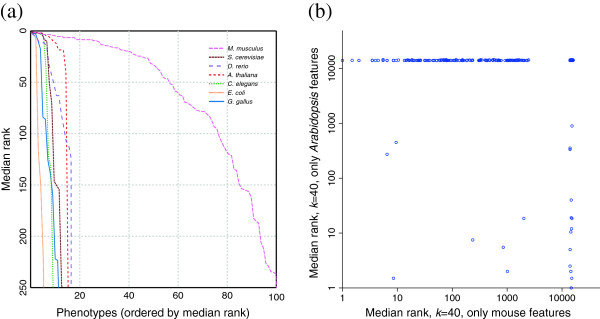
**Contributions by individual species to the prioritization of candidate genes. (a)** Each phenotype offers some sort of information for prediction of human disease genes. Mouse data seem to offer the most information about human diseases, as one would expect from the quality of the data and the proximity of the species in the phylogenetic tree. *Arabidopsis*, which is the furthest species from human in our database, unexpectedly provides as much information as mouse on top predictions, and is second at higher ranks. **(b)** This scatter plot demonstrates that the information offered by each species (in this case mouse and *Arabidopsis*) is highly independent, and suggests that integrating data from multiple species may be useful.

While similarity functions produced remarkably similar results, predictions coming from different species were much less strongly correlated (Figure 6), suggesting that weighting phenotypes by species in some manner may offer additional improvement. While each species provides uncorrelated prediction information, the human disease predictions are dominated by mouse whenever that species is included — likely because of the highly correlated nature of the exploration of gene–phenotype associations in mouse and human.

**Figure 7:**
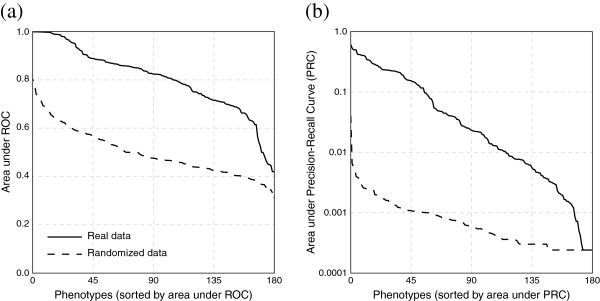
**Phenologs predict candidate genes substantially better than random.** Shown are **(a)** ROC and **(b)** precision–recall plots for *k*=100 naïve Bayes using the hypergeometric weighting function, predicting human (OMIM) gene–disease associations from human, mouse, worm, fruit fly, yeast, and plant gene–phenotype association data. We restrict the evaluation to only those phenotypes with four or more known genes. The solid line shows the actual data, and the dashed line shows the result on similarly sized random gene sets. Thus, integrating phenologs across multiple species successfully prioritizes candidate genes to an extent far greater than random chance.

Finally, we measured the extent to which our predictive performance was improved compared to random trials. To measure this enrichment, we generated a series of random gene-based matrices. For each phenotype-column of cardinality *p*, we marked *p* randomly-drawn genes as observed. We attempted to predict phenotypes-of-interest from these randomized matrices using our regular classifiers (Figure 7). (Note that we did not repeat the randomized matrix control for the orthogroup-based matrices, primarily because randomization of gene–phenotype associations eliminates the type of structure which made orthogroup-based matrices necessary.)

Importantly, we see a strong improvement in predictive performance on actual gene–phenotype associations as compared to randomized data. The method is able to recover all known genes for several real diseases — but is unable to recover withheld genes for any of the randomized diseases.

### Epilepsy

In addition to evaluating our method’s overall performance, we wished to take a closer look at its prediction of individual diseases in our database. We chose epilepsy because, despite offering ostensibly correct predictions, it actually scores somewhat poorly in cross-validation. In our initial three-fold leave-one-out test, only one of the three separately withheld genes was recovered at a reasonably testable rank (twelve, in this case).

**Figure 8:**
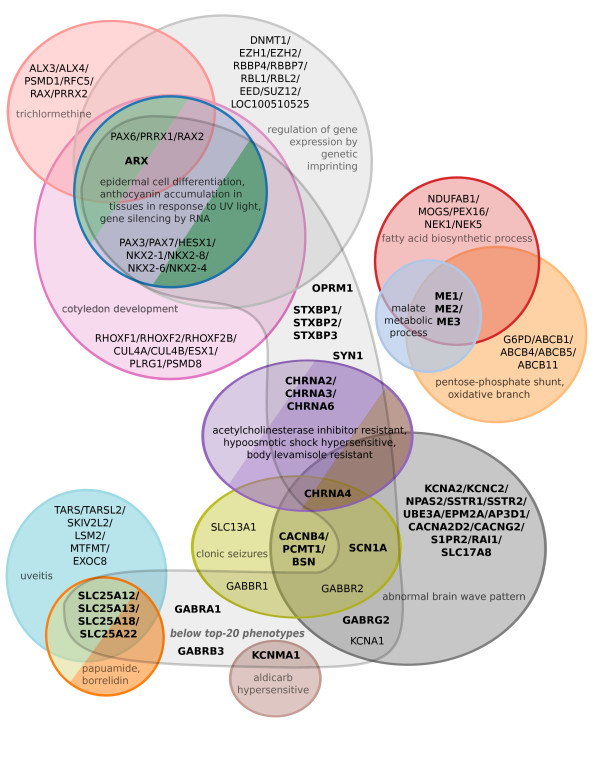
**A Venn diagram showing predictions for epilepsy based on the 40 most genetically similar phenotypes.** The analysis is primarily derived from *Arabidopsis*, yeast, worm, and mouse, based on the Pearson sample correlation, and using cosine similarity as the weighting function. The twenty closest phenotypes are each displayed separately, and the remaining twenty are aggregated into the category “below top-20 phenotypes.” Paralogs are grouped together when they coincide at a prediction score. Genes in bold represent the orthogroups used in the search — that is, those groups of orthologous genes where one or more paralog was already associated with epilepsy in our database. Colors correspond to those in Figure 9.

**Figure 9:**
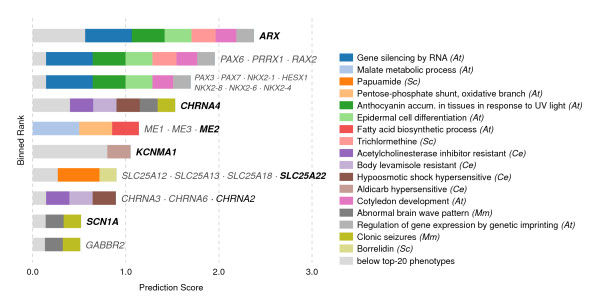
**Top candidate genes predicted for epilepsy.** Each row of this chart represents a set of genes predicted with the same score. If a gene symbol is printed in bold, it or a member of its orthogroup is already known to be involved. Rows with plain-text labels are novel predictions. The depicted search makes predictions based on the *k*=40 nearest neighbor phenotypes (from human, mouse, chicken, zebrafish, worm, yeast, and plant), and color codes the twenty nearest neighbor phenotypes’ contributions to each prediction (the remaining twenty-one are grouped in blue, as “below top-20 phenotypes”). The top scoring gene, *ARX*, is predicted primarily by Proud syndrome, hydranencephaly, and Partington’s syndrome, all of which are human diseases characterized partially by seizures; but information is also drawn from a variety of plant phenotypes. These predictions were generated using an additive classifier for ease of visualization. The distance function is Pearson sample correlation, using cosine similarity as the weighting function *w*.

Our method successfully identifies *GABBR1*, *GABBR2*[12], and *KCNA1*[13], which were absent from our database but known to be associated with the disorder. These were predicted primarily due to mouse phenotypes that resemble epilepsy (*clonic seizures* and *abnormal brain wave pattern*; Figures 8 and 9).

Top epilepsy predictions include *PAX6*, *PRRX1*, and *RAX2* (of which *PAX6* has been associated with seizures); and *PAX3*, *PAX7*, *HESX1*, and *NKX2-1*, *NKX2-4*, *NKX2-6*, and *NKX2-8* (Figure 9). Notably, *NKX2-1* is involved in mouse epilepsy [14], and *PAX3* appears in a region linked to the human version of the disease [15]); neither of these genes were in our database.

Interestingly, these predictions come from the *Arabidopsis* phenotypes *regulation of gene expression by genetic imprinting*, *cotyledon development*, *epidermal cell differentiation*, and *gene silencing by RNA*, as well as the yeast phenotype annotation for sensitivity to trichlormethine (nitrogen mustard, or *tris(2-chloroethyl)amine*).

To learn more about the general predictability of the epilepsy phenotype, we ran an expanded cross-validation, withholding each of the full set of 51 epilepsy genes in our database, and found that six genes could be predicted back — all within the top 120 ranks. We note that even when a phenotype performs poorly in cross-validation, it seems that our method still provides useful predictions.

We wanted to know the extent to which predictions could be attributed to paralogy (shared orthogroup membership) with genes already associated in our database with epilepsy. *GABBR1* and *GABBR2* are each singleton orthogroup members, and are thus independently predicted. *KCNA1* and *KCNA2* emerged as paralogs following the human–worm divergence, but are predicted from non-worm phenotypes — and are therefore also independent predictions.

*PAX6*’s plant–human paralogs make up the top three rank bins in Figure 9. We suggest that even non-independent predictions are of use, provided they are accompanied by independent predictions — since, as mentioned, *PAX3*, *NKX2-1*, and *PAX6* are all associated to some degree with seizures and/or epilepsy. Indeed, the inclusion of species in which these genes are not paralogs offers additional resolution on predictions and demonstrates the utility of our method.

### Predicting from *E. coli* — Pharmacologically-induced seizures

**Figure 10:**
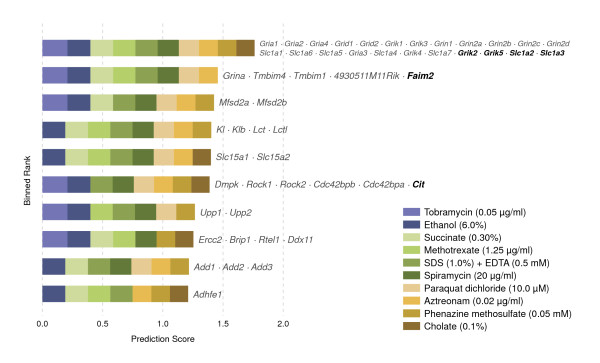
**Predicting mouse seizure genes from *E. coli *phenotypes.** These mouse phenotype predictions are constructed from the *k*=10 nearest neighbor *E. coli* phenotypes, using no other species. Predicting eukaryotic phenotype-linked genes from a prokaryote is necessarily coarse-grained, due firstly to evolutionary expansions of ancestral orthologs into larger orthogroups, and secondly to the tendency for some orthologs to vanish from certain species or become unrecognizable. Nevertheless, the probability of seeing an intersection of six or more orthogroups by chance, such as that between *sensitivity to tobramycin at 0.05 μg/ml* and the seizure phenotype, is 1.7×10^−4^ (without correction for multiple testing).

We then turned to a similar mouse phenotype, pharmacologically-induced seizures, to determine whether *E. coli* gene–phenotype associations could be used to make predictions about mammalian associations without additional information. We found that mouse genes linked to pharmacologically-induced seizures could be predicted extraordinarily well from *E. coli* alone in cross-validated tests: eight of the 48 genes associated with this mouse phenotype could be predicted back when withheld. These results are particularly impressive because they represent all six of the mouse–*E. coli* orthogroups associated with this seizure phenotype. Two of the orthogroups (*Grik2*/*Grik5* and *Slc1a2*/*Slc1a3*) are in the top prediction ranking bin; additionally, *Faim2* is in the top hundred ranks (Figure 10).

Next, we examined the predictions for promising new candidate genes. One of the most intriguing candidates was *α-adducin*, which is known to be reduced in the brains of rats experiencing kainate-induced seizures [16].

Another interesting prediction is *Sv2a* (*synaptic vesicle glycoprotein*). It was recently reported that a mutation in chicken *SV2A* leads to photosensitive reflex epilepsy [17]. Mouse *Sv2a* is a known binding site for levetiracetam, an antiepileptic drug [18], and *Sv2a*^−/−^ mice experience seizures and die within three weeks of birth [19,20].

We also examined the compounds associated with the source *E. coli* phenotypes to see if these were associated with seizures. The compounds included ethanol — alcohol poisoning and alcohol withdrawal symptoms include seizures — as well as paraquat, which causes seizures and brain damage in rats [21]; and aztreonam, which is a convulsant [22]. While many compounds might cause seizures if given in sufficient amounts, a control PubMed search for ten randomly chosen compounds associated with *E. coli* phenotypes in our database failed to turn up such clear associations.

Thus, both at the level of predicting candidate genes and affiliated compounds, the *E. coli* phenotypes appear to be relevant. Both of the genes discussed above may indeed represent reasonable new candidates for affecting pharmacologically-induced seizures and might warrant follow-up experiments. Finally, it is particularly striking that mammalian seizures — which are distinctly neurological phenomena — could be derived from processes so fundamental as to exist even in bacteria.

### Atrial fibrillation

We looked next at the human heart phenotype atrial fibrillation (AF), expecting to find that AF, like pharmacologically-induced seizures, was rooted in highly-conserved signaling defects. Instead, we found that the most predictive phenotypes were from mouse and chicken — quite unlike epilepsy, for which plants, worms, and yeast offered a great deal more information than mouse or chicken.

The AF phenotype performed well cross-validation in the gene-based configuration method. However, in the orthogroup-based cross-validation, only three of the eight genes associated could be predicted after being withheld. The removed genes were predicted at ranks 3–4, 15–16, and 81–94. Nonetheless, the novel predictions for this phenotype are worth noting.

**Figure 11:**
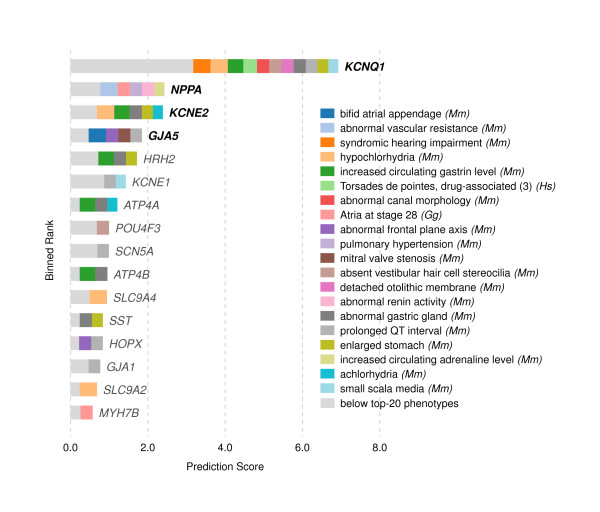
**The top candidate genes predicted for atrial fibrillation.** These predictions are constructed in the same manner as those in Figure 9. Limiting the search to *k*=40 neighbors in this case means that all predictive phenotypes come from mouse and chicken, though other species were included in the analysis. Interestingly, few of the informative mouse and chicken phenotypes are related to the heart in any obvious manner.

The top-ranked new prediction for atrial fibrillation (AF) is the *histamine receptor* H_2_ (*HRH2*), largely contributed by gastrointestinal phenologs in mouse (Figure 11). Histamine has been known to act on heart cadence for over a hundred years [23]. However, an empirical link between heart and gastrointestinal function was established by the recent observation that histamine increases the heart rate in pythons during digestion [24] — regulation which both occurs via the H_2_ receptor [25-27] and is apparently ubiquitous in vertebrates.

Similarly predicted are *ATP4A* and, further down the list, *ATP4B*, which are the *α* and *β* subunits of the H^+^/K^+^ ATPase. This proton pump is responsible for gastric acid secretion during digestion.

A somewhat speculative connection is offered by recent work, which showed cigarette smoke extracts cause an increase in the amount of H^+^/K^+^ ATPase in the stomach [28]. It is unclear — and worth testing — whether *ATP4A* and *ATP4B* are expressed in the heart. These genes could offer an additional route by which smoking contributes to heart problems.

Following *HRH2* and *ATP4A* is *HOPX*, or *homeodomain only protein x*, which is down-regulated during heart failure in humans [29]. It is not clear that *HOPX* is involved in AF per se, but again worth exploring in future experiments, as is the gene ranked next, *KCNE1*, based on orthologous phenotype *prolonged QT interval* — and seemingly also a factor in rare cases of atrial fibrillation [30-32].

*GJA1* (*gap junction protein, α*1, also known as *connexin 43* or *Cx43*) is one of the two most abundantly expressed connexins in the heart [33-35]. The other is *GJA5* (*connexin 40*), already associated in our database with AF. *Cx40* and *Cx43* seem to form heteromeric channels with different properties from homomeric channels [36]. *Cx43*, unlike *Cx40*, is essential for heart development and cardiac impulse conductance in mice [37]. Tuomi et al. observed that a dominant negative *Cx43* mutant causes severe AF [38]. Finally, atrial fibrillation was observed in a somatic mutation in human *GJA1*[39]. Notably, another channel similarly implicated was *SCN5A* (human cardiac sodium channel, voltage-gated, type V, *α* subunit). This sodium channel component has been associated with atrial fibrillation [40-42] but was missing from our database.

In terms of the predictor phenotypes themselves, the top AF phenologs can be grouped into three basic categories: cardiac, gastric, and auditory. We have explored the first two categories, but have not considered genes from the third. We note that while Jervell and Lange-Nielsen syndrome (i.e., long QT syndrome) has been associated with deafness for half a century [43-46] via alleles of *KCNQ1*[47] and *KCNE1*[48], other genes may yet be involved [49]. Further, Belmont et al. write of “a growing appreciation for conditions that affect hearing and which are accompanied by significant cardiovascular disorders” [50].

Given the success with which our method was able to predict AF genes — and with which it was able to identify potentially related disorders — exploration of additional candidates (e.g., *ATP4A*/*B*, *POU4F3*, and S1PR2) from Figure 11 may be warranted.

### Plant phenotypes — response to vernalization

Finally, having focused primarily on predicting mammalian phenotype- and disease-genes, we asked whether plant gene–phenotype associations could be predicted from the other species in our database.

Plants represented a particular challenge, since a number of factors reduce the specificity of predictions for plant phenotypes. Firstly, while human phenotypes are predicted at least in part from other mammals and even other vertebrates — which are phylogenetically similar — there are no close neighbor species to *Arabidopsis* in our database.

Secondly, while 19,439 of the 28,002 human genes in our database have orthologs in other species, the ratio is less promising for *A. thaliana* phenolog predictions: 12,668 of 27,325 have orthologs. The cause is likely again the lack of other plants in our database, compared to the several vertebrates from which to draw information for *H. sapiens*.

Third and finally, the *Arabidopsis* genome contains a great deal of redundancy, as observed in [51]: 37.4% of proteins belong to families of more than five members, compared to 12.1% in fruit fly and 24.0% in worm. In predictions that rely on gene orthology, as with phenologs, there is often no way to distinguish which of the plant paralogs is most relevant — except perhaps by relying on paralogous phenotypes.

Our orthogroup-based matrix formalism (Figure 2D) was thus critical for addressing the extensive divergence of gene families between distant species. In particular and as previously described, when attempting to predict *Arabidopsis* phenotypes, we noticed that the gene-based formalism resulted in asymmetric scores and unwarranted improvements in rank of certain predictions, particularly those where large orthogroups were involved (Figure 2A–C). The genes-as-rows configuration also inflated performance, as measured by ROC plots, during cross-validation — primarily due to the high frequency with which plant gene expansions co-participate in a biological process.

**Figure 12:**
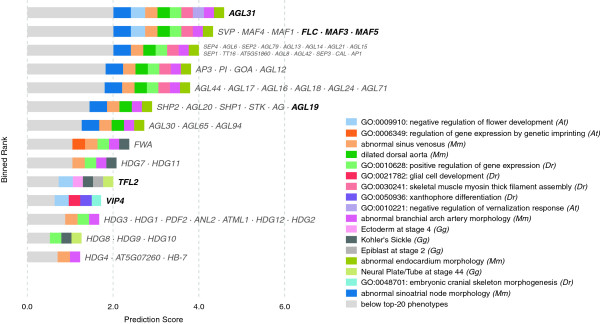
**Predicting performance of phenologs for plant phenotypes.** This figure mirrors Figure 6A, but demonstrates the prediction of *Arabidopsis* phenotypes from individual species (rather than human diseases from individual species). The red solid line shows the combined performance of predictions using all species except *Arabidopsis*. Yeast appears to be the most useful individual species for predicting plant phenotypes.

**Figure 13:**
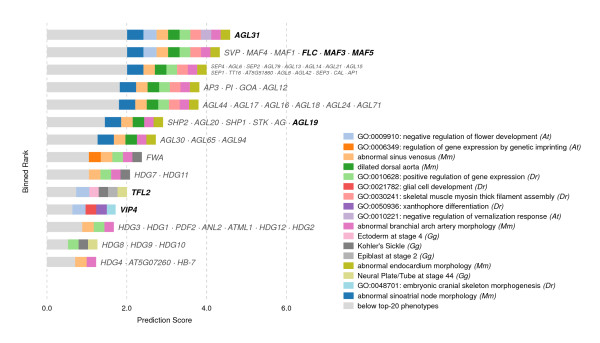
**The top candidate genes predicted for *Arabidopsis *response to vernalization.** Here, we demonstrate predictions for a plant phenotype, *response to vernalization*, while also demonstrating how including paralogous phenotypes may slightly enhance resolution. These predictions are drawn from phenotype data from each species in the database, with a neighborhood cutoff of *k*=40. Due to the large gene expansions in plants, as well as the relatively large distance of *Arabidopsis* from other species in our database, paralogs are often ranked together. In the first two bins, a large gene expansion is split into separate ranks by information from an *Arabidopsis* phenotype (which is paralogous rather than orthologous). Those ranks labeled with green text include at least one previously known vernalization response gene (that is, a gene that was already linked with vernalization response in our database).

Given this formalism, we then determined phenotypes which may be predictable by cross-validating predictions produced from all non-plant species in the database (Figure 12), and found a large number (greater than 50) of the plant phenotypes to be reasonably well-predicted based on non-plant phenotypes. We describe final predictions for the response to vernalization phenotype (Figure 13).

We selected this phenotype because it scores better than most other plant phenotypes in cross-validation; seven of the fifteen genes in this plant phenotype can be predicted back at low rank when withheld, representing two or three orthogroups (about half of the total number of orthogroups) depending upon the source species considered.

Although we cannot easily cross-validate predictions from paralogous phenotypes, since they are not sufficiently independent, we speculate that the inclusion of paralogous phenotype data may help to improve the specificity of the predictions at no perceivable cost.

Among the new candidate genes implicated, two are particularly notable. One of these, *EMF2* — which appears to be associated with vernalization-mediated flowering by its interaction with *CLF*[52] — is predicted based on seemingly unrelated orthologous mouse and human phenotypes (abnormal chorion morphology and endometrial cancer, respectively). *EMF2* is paralogous with known vernalization gene *VRN2*; however, it is ranked ahead of *VRN2* by its association with the related plant phenotype *negative regulation of flower development*. That *EMF2* was boosted by a potential paralogous phenotype supports the hypothesis that paralogous phenotypes are similarly useful to orthologous phenotypes in predicting gene function.

The second interesting prediction is *FWA* and its several paralogs (*HDG1–4*, *HDG7–12*, *PDF2*, *ANL2*, *ATML1*, *AT5G07260*, and *HB-7*). Certain *FWA* mutants produce a vernalization-insensitivity phenotype [53,54]. Candidates *ANL2* and *PDF2* both have late flowering phenotypes [55,56] markedly similar to that of *FWA*[57]. That discovery lends additional support for paralogous phenotypes, as neither *FWA* nor *PDF2* were associated in our database with regulation of flower development — but our method successfully identified *negative regulation of flower development* as a potential phenolog.

Empirically, we believe that the strength of our predictions for plant phenotypes are limited primarily by the quantity of gene–phenotype information available for plants. Notably, the addition of associations from other plant species would prove exceptionally useful for predicting not only *Arabidopsis* phenotypes, but crop species as well, and — as demonstrated earlier — even animal phenotypes.

### Datasets

We sought to determine whether the improvement in our method over the original Phenologs method could be attributed in part to the additional species information or arose exclusively from incorporating phenotypes beyond the nearest neighbor (as demonstrated in Figure 5).

**Figure 14:**
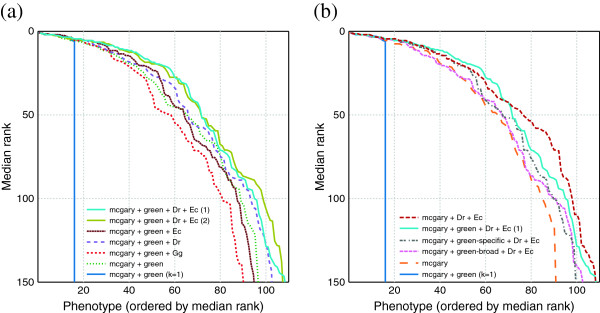
**Measuring the effect of additional datasets on predictive performance.** Here, we used our best classifier (naïve Bayes with Pearson sample correlation for a distance function, weighted by hypergeometric CDF), and subtract out datasets in order to determine their relative contributions. Unless otherwise indicated, classifiers were run with *k*=40. **(a)** demonstrates that for the original species used by McGary et al. (also including the new phenotypes from Green et al.), the *k* nearest neighbors method performs substantially better from the original Phenologs method (approximated by *k*=1). The datasets are labeled *mcgary* (mouse, worm, nematode, yeast, and plant), *green* (nematode), *Dr* for zebrafish, *Ec* for *E. coli*, and *Gg* for chicken. The best-performing analysis was repeated (labeled “(1)” and “(2)”, with different random test genes withheld) to demonstrate that performance is robust under cross-validation. **(b)** presents a test of whether specific phenotypes are more useful than broad phenotypes, by breaking down the *green* dataset into its components, *green–specific* and *green–broad*. We found that including both *green* datasets yielded the best results at relevant ranks, but that they both hurt results at less relevant ranks (beyond 45). Also shown is a comparison between the original datasets (*mcgary* alone) and the best-performing collection from **(a)**, with all datasets except chicken (represented by the solid cyan line).

We began by plotting the performance of those predictions drawn from the species used by McGary et al. (mouse, nematode, yeast, and plant, short-hand mcgary+green, notably including the additional phenotypes from Green et al.). As expected, we found that increasing *k* from 0 to 40 markedly improved the results (Figure 14).

Next, we tried adding the new species (chicken, *E. coli*, and zebrafish) individually, and found improvements at relevant ranks for *E. coli* and *D. rerio*. Surprisingly, inclusion of chicken data hurt the predictive performance. We also tried adding *E. coli* and *D. rerio* at the same time, and saw an additional increase in performance.

Given the decrease in performance resulting from the inclusion of chicken phenotypes, we sought to determine whether the additional *C. elegans* datasets were negatively affecting performance. In Figure 14B, we tried leaving out the broad and specific components of the *green* dataset. We found that either component alone performed worse than the combination. We also tried leaving out the *green* datasets altogether, and found that their inclusion moderately improved performance at relevant ranks, but decreased performance beyond around rank 45.

These mixed results with the datasets are somewhat surprising. We expected that the *in situ* hybridization expression annotations from GEISHA would be useful for human predictions not only because gene expression stage and location should correlate highly with phenotype, but also because chicken — like mouse — is more closely related to human compared to other species in our database.

The distributions of genes per phenotype for human, mouse, *Arabidopsis*, and chicken were similar (not pictured). Only *E. coli* differed substantially, with most phenotypes involving between 800 and 1,000 genes. However, in general, the counts of *E. coli*–human *orthologs* involved in bacterial phenotypes are much smaller due to the relatively small fraction of genes with orthologs between the two species.

## Conclusions

In summary, we set out to improve upon the results of the original phenolog project by unifying information from a “neighborhood” of phenotypes surrounding the phenotype or disease of interest. Our method produces ranked predictions for a large percentage of human diseases in OMIM, as well as for plant biological process-based phenotypes.

Notably, we were able to demonstrate the correct prediction of at least one gene associated with the mouse phenotype *pharmacologically-induced seizures* using only phenologs from *E. coli*. While McGary et al. demonstrated the existence of deep homology between mice and single-celled eukaryotes, our work suggests that examples of deep homology exist — and may even offer useful predictions — between prokaryotes and eukaryotes.

We also demonstrate that the term “phenotype” may be interpreted broadly when incorporating gene-association data for phenolog-based predictions. Gene Ontology biological processes are one potential source. Another potential source is annotations for *in situ* hybridization experiments, such as GEISHA, but it may be necessary to refine such a phenotype database by hand.

Finally, we give a number of concrete gene predictions for the human diseases atrial fibrillation and epilepsy, and show how phenologs may be used to generate hypotheses and a biological context that correctly connect categories of diseases, such as disorders of the heart, stomach, and sensorineural system.

## Methods

### Cross-validation

For the gene-based matrix, we compared classifiers and metrics using *n*-fold cross-validation, and calculated receiver operating characteristic (ROC) and precision–recall curves for each disease or phenotype to be predicted. Classifiers could be represented by arrays of area-under-the-curve measurements.

With the orthogroup-based matrix we chose a simpler and faster “leave-one-out” cross-validation scheme, where one observed gene association was hidden for each disease. Noting that some orthogroups have multiple genes associated with the same phenotype, we also hid any orthogroups associated with hidden genes. Since a gene may be part of one orthogroup for each species included in the search, we measured the rank of predicted genes rather than predicted orthogroups. When multiple genes were predicted with the same score, the mean rank was used.

The leave-one-out procedure was repeated three times for each phenotype, taking the median hidden gene rank to be representative of the classifier–phenotype performance.

### Additional phenotype data

In addition to those databases described in [9], we incorporated orthology and gene–phenotype data from a variety of additional species. We excluded any phenotypes with fewer than three associated genes.

Our choice of species was driven primarily by availability of data in a useful format, namely that phenotype annotations could be expressed qualitatively, and that we we could link those annotations to a protein sequence; for example, we wished to incorporate phenotype data from the agricultural plant database Gramene, but most or all phenotype-associated genes lacked sequences.

Human diseases came from OMIM as for [9], but updated on August 17, 2011. Additional *C. elegans* phenotypes came from Green et al. [58] and were broken down into two datasets, *green–broad* and *green–specific*, according to the classifications given by the authors in the second supplemental table, “Broad Phenotypic Category” and the more specific subcategories into which they were divided. We chose to maintain the dual categorization primarily because a number of the more specific phenotypic classes were monogenic, and hence would have been ignored altogether in our analysis.

*E. coli* phenotypes were taken on May 20, 2011, from the file ‘coli_FinalData2.txt’ [59]. Each gene’s phenomic profile was sorted by score, assigning both the top and bottom forty conditions to the gene. Thus, each condition was considered to be a phenotype, and the genes associated with that phenotype were those genes whose growth was most affected — either positively or negatively — in the corresponding condition.

We considered fruit fly phenotypes from FlyBase [60]. Unfortunately, FlyBase’s dataset — while extensive — makes use of a controlled vocabulary optimized for manual searching rather than high-throughput analysis. The only way to connect a phenotypic class annotation to an anatomical location or developmental stage is by allele and literature reference — if these are given at all. We attempted to match anatomical annotations for mutant phenotypes to annotations from the *phenotypic class* ontology, joining on allele and publication. While it was possible to predict some human diseases based on fruit fly phenotypes from FlyBase, the results were noisy and difficult to interpret, and we ultimately chose to exclude fruit fly results.

Zebrafish phenotypes consisted of gene ontology (GO) biological processes from ZFIN [61], keeping only those annotations with evidence types of *IMP*, *IDA*, *IPI*, *IGI*, *TAS*, *NAS*, *IC*, and *IEP* — the same procedure used for *Arabidopsis* phenotypes, obtained from TAIR [62]. These evidence types were selected so as to avoid the inclusion of annotations that originated directly from knowledge of other model organisms.

For chicken (*Gallus gallus*) phenotypes, we utilized *in situ* hybridization annotations from GEISHA [63], kindly provided in XML format on June 24, 2011. If there were more than fifty genes associated with a specific location and more than three at a specific state at that location, a new phenotype was created (“*anatomical location* at stage *x*”); and regardless, each location became an independent phenotype. We defined phenotypes as gene–expression associations in specific anatomical locations. For those locations with more than fifty genes annotated, we created additional phenotypes for each stage with greater than three associated genes.

## Availability of supporting data

The data sets supporting the results of this article are available at http://www.phenologs.org/knn/.

## Competing interests

The authors have applied for a patent on the Phenologs method discussed in McGary et al., 2010, but otherwise declare that they have no competing interests.

## Authors’ contributions

JOW and UMSB contributed equally, analyzing the data and assembling the datasets, with the assistance of JL and KM, under the supervision of EMM, JOW, UMSB, and EMM wrote and edited the manuscript. All authors read and approved the final manuscript.
